# Associations between adolescent students’ multiple domain task value-cost profiles and STEM aspirations

**DOI:** 10.3389/fpsyg.2022.951309

**Published:** 2022-12-22

**Authors:** Janica Vinni-Laakso, Katja Upadyaya, Katariina Salmela-Aro

**Affiliations:** Minds Hub Research Group, Faculty of Educational Sciences, University of Helsinki, Helsinki, Finland

**Keywords:** task values, cost, self-concept, gender, STEM aspirations, latent transition analysis

## Abstract

According to the modern expectancy-value theory, students’ task values may differ across domains, manifesting as varying motivational patterns. In middle school, students’ motivation becomes increasingly apparent and may direct their future occupational aspirations. Using a person-oriented approach, this study examines students’ self-concept, and positive and negative task values (i.e., utility value, intrinsic value, and emotional cost) across Finnish language, math, biology, and physics, and the stability of the identified profiles. Further, the associations of the profiles with students’ subsequent academic achievement and math and natural science, technology, engineering, and mathematics (STEM)/health science STEM aspirations, and gendered effects were examined. Longitudinal data was collected through Grades 7 to 9 in 21 middle schools in Helsinki, Finland (*N* = 1,309, *N* = 1,179, *N* = 818, respectively; age 13–15 years; 55.9% female). Latent profile analysis (LPA) identified four task value profiles in Grades 7 and 8: *Low motivation high cost STEM* (13%/13%) showed low task values with high cost, especially in math and physics; *High motivation low cost STEM* (7%/8%) showed the highest task values with the lowest cost, especially in math and physics; *High motivation high cost* (18%/17%) showed high task values and cost across domains; and *Moderate motivation and cost* (62%/62%) showed moderate task values and cost across domains. The latent transition analysis identified *Moderate motivation and cost* as the most stable profile across 2 years. In comparison to the other profiles, students with a *Low motivation high cost STEM* profile were less likely to have STEM aspirations in Grade 9. These results suggests that majority of middle school students are highly to moderately motivated in various domains, however, some students simultaneously experience high cost. It may reflect the increasingly difficult courses and study demands in middle school.

## 1 Introduction

Globally, there is a topical concern focusing on the increasing mismatch between the growing need for skilled labor in science, technology, engineering, and mathematics (STEM) fields and the low appeal of these areas of study and their related careers for youth (Tytler, 2014; Martin et al., 2016; OECD, 2016). In particular, attracting women and minorities to STEM-related fields has been challenging (Homer et al., 2014; National Science Foundation [NSF], 2017). Boosting STEM studies and careers among both women and men is required in order to build a more skillful workforce that is responsive to future labor market needs. In addition, researchers, educators, and policymakers should help narrow the gender gap in the STEM fields, as their actions could have multiple effects that would improve society as a whole. Ensuring that both women and men are better equipped to secure steady and well-paid jobs would ensure social mobility, advance STEM research and innovation, and reduce the risk of social exclusion for women and minorities. Many (inter)national initiatives and programs have been pursued to enhance this goal by increasing awareness of the education and career possibilities in the STEM fields and enhancing students’ motivation in science (e.g., UNESCO, 2020). To understand students’ educational and occupational choices, and the gendered effects, researchers have also studied the formation and development of students’ science motivation and their aspirations in STEM education and careers (see, e.g., Potvin and Hasni, 2014; Wang and Degol, 2017 for reviews). In particular, the research addressing the roles of students’ self-concept, interest, expectations, and achievement as the main contributors to STEM aspirations has gained a vast amount of scientific attention (e.g., for review see Watt, 2016; Guo et al., 2018; Kang et al., 2019). Utilizing a number of these constructs, we study the formation and constancy of students’ task value profiles and how they predict subsequent STEM aspirations.

### 1.1 Expectancy-value theory

In this study, we draw on the expectancy-value framework (Eccles et al., 1983) to investigate students’ task motivation in middle school. According to the expectancy-value theory, students’ motivation can be divided into ability beliefs and expectancies, and subjective task values (Eccles and Wigfield, 2020). Ability beliefs and expectations relate to questions such as “Can I do this task?” (referred to here as domain specific self-concept), whereas subjective task values provide an answer to questions such as “Do I want to do this task?” Both aspects of motivation are important, as they are often associated with student achievement as well as achievement-related choices and career aspirations (for reviews see Wigfield and Cambria, 2010; Watt, 2016). However, task values are particularly important for student achievement and learning: regardless of their self-concept, a student may not engage with learning or accomplish different tasks if they do not also value the subject or activity (Ryan and Deci, 2000). Task values are further divided into intrinsic, attainment, and utility values and costs. Intrinsic value refers to students’ subjective interest and the inherent enjoyment they experience when involved in a task (Eccles and Wigfield, 2020). Attainment value describes the importance of doing well in a given task, and utility value refers to the task’s future relevance, or how demonstrating competence in the current task/domain will benefits one’s future aspirations or career (Eccles and Wigfield, 2020).

The costs, in turn, are divided into the following categories: the demands associated with investing the significant effort required to succeed in a task (effort cost), the choices involved in setting aside other interesting/useful/important options in order to engage in a task (opportunity cost), and the psychological experiences (e.g., emotional exhaustion or stress) related to learning or completing a task (Wigfield and Eccales, 2020). To date, task value research has primarily focused on the positive values, and the perceived costs have been neglected (Flake et al., 2015), especially in longitudinal settings (Wigfield and Cambria, 2010). Positive task values and self-concept typically promote student motivation while perceived costs have been identified as a hinderance (Barron and Hulleman, 2015) in the same domain. High costs have been associated with low self-concept (e.g., Vinni-Laakso et al., 2019), interest, and academic achievement (Perez et al., 2014; Barron and Hulleman, 2015; Flake et al., 2015). High perceived costs may also lead to procrastination, avoidance behavior (Jiang et al., 2018), and impaired psychological academic wellbeing (Watt et al., 2019; Tuominen et al., 2020). Somewhat controversially, several studies have positively associated perceived cost with positive task values (Gaspard et al., 2019; Lee et al., 2022). This finding implies that high positive task values, aka high motivation, can increase the effort a student will expend in their studies, and/or they might be more willing to engage in a particular task over other valued alternatives. Emotional cost may also accompany high motivation: the stress associated with academic achievement in a given domain potentially leads to a student placing a high value on that domain. Thus, cost in the expectancy-value model could be more complex than previously assumed (Eccles et al., 1983), and it may uniquely contribute to student motivation (Barron and Hulleman, 2015).

This study aims to clarify the role of cost in student motivation by examining task value patterns in middle school. We focus on emotional cost with self-concept, intrinsic value, and utility value to gain an understanding of how negative emotional experiences interact with positive task values and contribute to motivation profiles across four domains: Finnish language, math, biology and physics.

### 1.2 Motivation profiles and stability

The decline of student motivation in science and mathematics has been identified in large-scale assessments, such as the Program for International Student Assessment (PISA) and Trends in International Mathematics and Science Study (TIMMS) (Martin et al., 2016; OECD, 2016). However, studies that methodologically examine student motivation only at the mean level cannot capture individual differences between students nor identify possible subgroups. Therefore, a number of studies have employed person-oriented approaches to research students’ motivational beliefs across domains and reveal their study-related task value patterns. Prior research has found relatively similar profiles using variety of positive task value facets, namely interest, utility value and attainment value with self-concept. The profiles found often reflect high motivation with high self-concept and task values, moderate motivation with moderate self-concept and task values, and low motivation with relatively low self-concept and task values in all domains, but also mixed motivation with high self-concept and task values in some domains that are accompanied by low self-concept and/or task values in other domains (e.g., Chow and Salmela-Aro, 2011; Viljaranta et al., 2016; Guo et al., 2018; Lazarides et al., 2021; Oppermann et al., 2021). From here on, we use the terms high/moderate/low motivation or mixed motivation in profile names to refer to the relative levels of self-concept and interest/utility/attainment value in the studied samples. However, these studies have only examined positive task values and self-concept, and they have excluded cost. The few cross-sectional studies that have examined motivation patterns with cost in math have identified different profiles of students’ motivation and cost; these studies have depicted high/low success expectations, utility values, and cost (Hodis and Hodis, 2020), which have reflected overall differences (e.g., low, average, high motivation) in students’ task values and cost profiles (see also Fryer and Ainley, 2019). Only a few studies have identified more specific nuances in students’ motivation profiles when low motivation is associated with high cost (Gaspard et al., 2019; Watt et al., 2019; Lee et al., 2022). These results have indicated that while some students with high cost will disengage from school and learning, other students may in fact orient toward high academic achievement (Conley, 2012; Watt et al., 2019; Tuominen et al., 2020). Moreover, studies that included positive and negative value beliefs (i.e., cost) also identified a moderate motivation profile that is characterized by an average level of task values and cost (Gaspard et al., 2019; Perez et al., 2019; Watt et al., 2019; Lee et al., 2022). This finding implies that costs do not function in isolation, and students may simultaneously consider that a domain is interesting and useful while engaging in study and experiencing the costs.

The person-oriented studies that have included costs have generally focused on specific domains, such as math (Watt et al., 2019; Hodis and Hodis, 2020), science (Perez et al., 2019; Watt et al., 2019), chemistry (Lee et al., 2022), or language (Fryer and Ainley, 2019). One study (Gaspard et al., 2019) that did examine task value-cost profiles across math and English as a second language identified two profiles characterized by mixed motivation (i.e., High language/Low math, Low language/High math) and two profiles with overall motivation (i.e., High motivation in language/math and Moderate motivation in language/math). The study found that perceived cost was positively associated with positive task values and self-concept in both domains, which resulted in the high motivation profile simultaneously indicating high cost. The finding showed that in contrast to the theoretical hypothesis of expectancy-value theory, cost does not merely serve as a barrier to motivation; instead, the hierarchical task values and cost together form the personal motivation patterns observed in different domains (Barron and Hulleman, 2015).

To understand students’ educational choices and the factors that influence them, it is essential to first examine the formation of students’ nuanced task value patterns during their middle school years, as during this period, task motivation begins to play a more important role in their studies. Prior research has shown that motivation profiles remain moderately stable over time (e.g., Fryer and Ainley, 2019; Lazarides et al., 2019; Oppermann et al., 2021), whereas some studies have found that profile memberships reveal noticeably clear changes (e.g., Lazarides et al., 2021). To the best of our knowledge, longitudinal person-oriented studies that include task values and cost remain unexplored. Additional research is required to gain an understanding of the stability of students’ task value-cost patterns in middle school. Therefore, the aim of this study is to examine longitudinal profiles in students’ task values and cost in Finnish language, math, biology, and physics in Grades 7 and 8 of middle school. In the expectancy-value literature, native languages and math have received extensive research attention as the stereotypical female and male domains (for a review see Wigfield and Eccales, 2020), whereas studies considering physics and biology are more recent and scarce. In order to examine STEM aspirations, three STEM-related domains were selected with Finnish language to project the findings of this study to the prior findings, and to examine stereotypically gendered motivational beliefs across domains.

### 1.3 Task motivation, achievement, and STEM aspirations

Expectancies and values often predict students’ school achievement and direct their educational choices (Bong, 2001; Guo et al., 2015) and occupational aspirations (Chow et al., 2012; Guo et al., 2015, 2017, 2018). For example, high expectancies and/or self-concept and task values in math, physics, and chemistry predict students’ entry to STEM education programs and further occupations in STEM fields (Bong, 2001; Jiang et al., 2020; Wille et al., 2020). In particular, students’ math interest and utility values in middle school are associated with their choice of STEM major when enrolling in higher education (Guo et al., 2015). Similar results have also been reported for math-intensive STEM majors (Wille et al., 2020). In addition, science interest already appears to be relevant in the formation of elementary students’ occupational STEM aspirations (Vinni-Laakso et al., 2019). Cost has also been shown to influence adolescent students’ academic behaviors and outcomes. High cost is associated with lower academic performance in higher education (Flake et al., 2015) and contributes to increased intentions to withdraw from a STEM education/major in college (Perez et al., 2014). In middle and high school, high perceived cost was associated with students’ adoption of avoidance goals, negative classroom affect, procrastination, intentions to divert from studying, and achievement in mathematics (Jiang et al., 2018; Jiang and Rosenzweig, 2021).

Rather than showing uniformly high levels of task values, the patterns of student motivation vary in terms of academic achievement and educational choices and reveal task values with intraindividual hierarchies that contribute differently to students’ decisions and choices (Eccles and Wigfield, 2020). Task motivation patterns may affect students’ academic achievement through the educational levels (Eccles et al., 1993; Wigfield et al., 1997; Guo et al., 2017) and further guide their educational choices and aspirations (Perez et al., 2014; Guo et al., 2015; Jiang et al., 2020). The students with high task values in math and science and low task values in other domains are more likely to pursue STEM fields than students with high task values in all domains in elementary (Oppermann et al., 2021) and secondary school (Chow et al., 2012; Guo et al., 2018; Gaspard et al., 2019). There has been less research, however, examining how perceived costs relate to students’ motivational patterns and shape STEM pathways. The few studies that have investigated patterns of self-concept, positive task values, and cost have shown that these motivational constructs are associated with academic outcomes (Gaspard et al., 2019; Perez et al., 2019; Lee et al., 2022). Middle school students that were identified in mixed motivation profile as having a high math and low language motivation were more likely to aspire to a STEM major in college in comparison to other profiles that showed either high or moderate motivation across domains or high language and low math motivation (Gaspard et al., 2019). Similarly, college students’ motivational patterns were associated with their academic achievement (Perez et al., 2019; Lee et al., 2022). In comparison to students with a very high motivation and low cost profile or a high motivation and moderate cost profile, students grouped in the moderate motivation profile with moderate self-concept, task values, and cost demonstrated lower achievement and completed fewer courses in the same academic year and also after 4 years. Significantly, these studies assessed opportunity cost and effort cost instead of emotional cost, which is the focus of the current paper.

There is currently a void in the literature of emotional cost and how it shapes task motivation and students’ academic performance and outcomes. As opportunity and effort costs, also emotional cost has found to be negatively related to interest, utility and attainment value (Barron and Hulleman, 2015; Flake et al., 2015). However, emotional cost as a psychological factor relates more closely to emotion regulation and wellbeing (e.g., stress, exhaustion, anxiety), whereas opportunity cost and effort, where students evaluate how much time and effort they need to or are willing to put on a task/domain in order to succeed, are not emotionally draining. As shown, for some students high utility value and attainment value are accompanied with high emotional cost (Watt et al., 2019; Tuominen et al., 2020) and may have detrimental consequences in students’ psychological academic wellbeing. It is important to bear in mind that emotional costs in academic setting may contribute to developing burnout symptoms which in turn may lead to lower academic achievement, lower educational aspirations, and even drop-out in later education (Salmela-Aro, 2017). In order to understand the role of emotional cost in task motivation and to identify possible vulnerable groups, we need to examine patterns of positive task values simultaneously with emotional cost. Here, we follow the theoretical framework in which intraindividual hierarchies of expectancies and task values across domains direct students’ academic choices. In this study, we examine how middle school students’ self-concept, interest, utility value, and emotional cost in the domains of Finnish, math, biology, and physics function together to predict students’ academic achievement and occupational STEM aspirations. It is crucial to investigate students’ motivational patterns in middle school in order to understand how they direct students’ achievement choices in the transition to higher secondary education.

### 1.4 Gendered differences in science motivation and STEM aspirations

Studies have found gendered differences in students’ task values and achievement across domains, and most frequently in languages, math, and science. It has been shown that, in comparison to girls, boys generally report higher self-concept and task values in math and science; however, girls have been shown to report higher self-concept and task values in verbal domains (e.g., Jacobs et al., 2002; Nagy et al., 2008; Watt et al., 2012; Gaspard et al., 2015; Guo et al., 2015). In general, girls show higher academic achievement across domains (Watt, 2016). Moreover, gender differences often occur in task motivation patterns, which show that in math and science, girls typically belong to the low motivation profile while boys often have a high motivation profile (Chow and Salmela-Aro, 2011; Guo et al., 2018; Gaspard et al., 2019; Oppermann et al., 2021). In addition, studies have shown that boys often report more STEM aspirations than girls (Eccles, 2011; Wang and Degol, 2013). Recently, researchers have begun to broaden the traditional STEM categories to include the math and natural sciences (incorporating physical science, technology, engineering, and mathematics) as well as the life sciences and medical sciences (see Dicke et al., 2019; Toh and Watt, 2022). Previous research has shown that girls aspire to the life science occupations more often than boys, whereas boys are more likely to express an interest in math and natural science occupations (e.g., Dicke et al., 2019; Oppermann et al., 2021; Toh and Watt, 2022). The recent STEM categorization of the math and natural science and health science domains offers a way to examine the nuanced gendered pathways toward STEM careers.

From this standpoint, the present study investigates the patterns and stability of students’ task values and cost across multiple domains and their connection to later academic achievement and STEM aspirations. By focusing on both the positive and negative task values across domains, this study aims to clarify how task values and emotional cost are associated among individual students and how they form domain specific motivation patterns. In addition, this study examines the possible gendered differences in students’ motivational patterns, academic achievement, and STEM aspirations.

## 2 The current study

### 2.1 The finnish education context

In Finland, students complete 1 year of compulsory kindergarten before they start school in the year they turn 7. Elementary education covers Grades 1–6, after which students enter middle school (Grades 7–9). All of the domains in middle school have a subject teacher, whereas the lower Grades 1–6 are taught by a homeroom teacher. Students in Finland are directed into a specific study path in Grade 9 when they are 16 years of age, which is relatively late compared to many other countries. The choices for secondary education follow students’ educational aspirations by directing them into an academic track, a vocational track, or both. The selection of students for each school is based on students’ preferences and their grade point average (GPA). In addition, when students enter high school, they need to select either the basic math track or the advanced math track, which differ in terms of the number of courses and the level of difficulty. This choice creates a critical filter for further STEM education, as without completing the advanced math studies in high school, students’ options to apply for university STEM programs are limited. Thus, it is worthwhile to investigate students’ task values in middle school as relevant antecedents for educational choices in high school.

### 2.2 Objectives

Research question 1: What motivational profiles can be identified in Grades 7 and 8 according to the level of students’ interest and utility value, self-concepts of ability, and cost in Finnish language, math, biology, and physics?

Hypothesis 1: We expected to find four motivation profiles: a high motivation profile characterized by high positive task values, and self-concept in all domains (e.g., Viljaranta et al., 2016; Gaspard et al., 2019; Lazarides et al., 2021; Oppermann et al., 2021); a low motivation profile with low positive task values, and self-concept across domains; a mixed motivation profile with high positive task values, and self-concept in math and physics and low positive task values, and self-concept in Finnish (Oppermann et al., 2021); and finally, a moderate motivation profile with average positive task values, and self-concept across domains (Gaspard et al., 2019; Perez et al., 2019). Based on the few prior studies that have addressed cost, we expected that for some students, high motivation may accompany high cost (Watt et al., 2019; Tuominen et al., 2020; Lee et al., 2022). As there is a lack of previous empirical studies, the research examining the role of cost in students’ cross-domain motivation profiles was exploratory.

Research question 2: To what extent do students’ profile memberships change from Grade 7 to 8?

Hypothesis 2: Based on prior research, we expected the motivational profiles to be somewhat stable from Grade 7 to 8 (e.g., Lazarides et al., 2019; Oppermann et al., 2021). However, our hypotheses about the stability of motivational patterns were tentative given the lack of systematic longitudinal research simultaneously examining self-concept, positive task values, and cost in multiple domains.

Research question 3: Do students’ motivational profiles differ in terms of their subsequent academic achievement?

Hypothesis 3: We expected that a high motivation profile with high positive task values and self-concept and high or low cost would be associated with the highest academic achievement (Gaspard et al., 2019). In addition, we expected that a low motivation profile with low positive task values and self-concept would reflect the lowest academic achievement and be clearly differentiated from other profiles (Perez et al., 2019). However, given that prior studies have rarely simultaneously researched self-concept, positive task values, and cost in multiple domains, our hypotheses regarding motivational patterns predicting achievement remained tentative.

Research question 4: To what extent do the identified motivational profiles differ in terms of students’ STEM aspirations?

Hypothesis 4: We expected that a high motivation profile with high positive task values and self-concept across domains and/or high motivation in math and physics (e.g., Chow et al., 2012; Guo et al., 2018; Oppermann et al., 2021) would be associated with the highest occupational STEM aspirations. Again, our hypotheses about the joint cross-domain motivational patterns predicting STEM aspirations were empirical.

Research question 5: Do students’ motivational profile memberships, academic achievement, and STEM aspirations differ in terms of gender?

Hypothesis 5: We expected that girls would be more likely to have a high motivation profile with high positive task values and self-concept across domains (e.g., Chow et al., 2012; Watt et al., 2019; Oppermann et al., 2021) while boys would be more likely to have a low motivation profile across domains (Watt et al., 2019; Oppermann et al., 2021) and/or a math-motivated profile (Chow et al., 2012; Guo et al., 2018; Oppermann et al., 2021). We also expected girls to show higher academic achievement across the measured domains (Watt, 2016) and have more health science STEM aspirations than boys, and we expected boys to report more math and natural science STEM aspirations than girls (Dicke et al., 2019; Toh and Watt, 2022).

## 3 Materials and methods

### 3.1 Participants and procedure

The data was collected from students in Grades 7–9 (*N* = 1,309, *N* = 1,179, *N* = 818, respectively; age 13–15; 55.9% female) in a total of 21 middle schools in the Helsinki metropolitan area during the spring semesters of the years 2014–2016. Population in Finland is homogeny regarding the racial variation where 5% of the population had a foreign background in year 2021 (Suomen Virallinen Tilasto [SVT], 2022).^1^ Moreover, families’ socioeconomic (SES) variation is minimal as low income families are supported by social welfares. Thus, collecting information on family’s SES from students’ is challenging, resulting that the data only include students’ self-report information of their parent working/not working. Snowball sampling strategies were used to include new students and schools each year. Students filled in paper-based self-reports during class. Active parental consents were obtained from all participating students. The Education Division of the city of Helsinki pre-examined the research plan and gave permission to conduct the study.

### 3.2 Measures

#### 3.2.1 Subjective task values

An adapted task value scale (Eccles et al., 1983) was used to assess students’ subjective task values and included *Utility value* (“*The subject is useful*”), *Interest* (“*The subject is interesting*”), *Self-concept* (“*I am good at the subject*”), and *Cost* (“*The subject exhausts me*”) for Finnish language, mathematics, biology, and physics on a seven-point Likert scale (1 = Not at all, 7 = Very much). Scale reliability estimates (i.e., Cronbach’s alpha) cannot be provided because of the one-item measure for the subjective task values.

#### 3.2.2 Occupational aspirations

In the third data collection wave, students’ occupational aspirations were measured with an open-ended question: “What kind of work would you like to do when you grow up?” The students’ responses were first coded into occupational fields based on International Standard Classification of Occupations, 2008 (ISCO-08) endorsed by the Governing Body of the International Labor Organization (ILO). These classifications were then further divided into (1) non-STEM, (2) health science occupations, and (3) math and natural science occupations including engineering and ICT following the OECD STEM classification used in OECD (2016) (see Results, Annex A1). We used these classification criteria based on the field of occupation, and did not divide students occupational aspirations by the level of education (professional and assistant). As an exception for ISCO-08 coding, a psychologist was considered as a health profession and categorized as health and medical science occupations not as a law/culture/social sciences. Students most frequent answers coded as Math and natural science STEM were an architect, an engineer, and a programmer, whereas the most frequent occupations coded as Health science STEM were a doctor, a veterinarian, psychologist, and a nurse. The most frequent answers coded as non-STEM occupation were a lawyer, a teacher, an entrepreneur, a pilot, a police officer, a dancer, and an actor (see Appendix for the full list of named occupational aspirations). We admit that STEM categorization were in some cases ambiguous (for example a researcher can be in the various fields but are here coded as math and natural science STEM), and sometimes students answers were difficult to interpret as in the case “something related to art.” The encoding followed the coding scheme and was completed by two persons separately. The majority of the responses (*N* = 413) were coded as non-stem occupations (*n* = 257; 62.1%) while 27.5% of the responses were coded as health science STEM (*n* = 114) and only 10.4% of the responses as math and natural science STEM (*n* = 43). Based on these classifications, three dummy variables were created: (1) Math and natural science STEM vs. other fields; (2) Health science STEM vs. other fields, and (3) Combined STEM including both math and natural science and health science STEM vs. other fields (see Table 2).

**TABLE 1:** Descriptive data and correlations of the study variables.

	Grade 8															
	Finnish				Math				Biology				Physics			
	Utility	Interest	SC	Cost	Utility	Interest	SC	Cost	Utility	Interest	SC	Cost	Utility	Interest	SC	Cost
Grade 7																
Finnish utility		0.52**	0.38**	–0.05	0.41**	0.18**	0.12**	0.10**	0.42**	0.22**	0.23**	0.03	0.34**	0.16**	0.13**	0.07*
Finnish interest	0.49**		0.54**	−0.17**	0.20**	0.29**	0.14**	0.03	0.30**	0.35**	0.27**	0.01	0.25**	0.29**	0.22**	–0.03
Finnish SC	0.42**	0.53**		−0.26**	0.23**	0.25**	0.40**	–0.05	0.22**	0.25**	0.43**	–0.05	0.20**	0.19**	0.36**	–0.04
Finnish cost	−0.19**	−0.31**	−0.35**		0.01	–0.01	–0.05	0.53**	0.05	–0.01	−0.07*	0.57**	0.07*	0.07*	–0.03	0.52**
Math utility	0.34**	0.21**	0.27**	–0.05		0.50**	0.40**	−0.12**	0.44**	0.26**	0.26**	–0.02	0.59**	0.32**	0.32**	−0.06*
Math interest	0.21**	0.34**	0.24**	–0.05	0.51**		0.71**	−0.36**	0.31**	0.44**	0.35**	−0.09**	0.43**	0.62**	0.52**	−0.21**
Math SC	0.15**	0.15**	0.36**	−0.06*	0.38**	0.66**		−0.40**	0.20**	0.27**	0.45**	−0.08**	0.33**	0.47**	0.66**	−0.23**
Math cost	–0.05	–0.05	−0.08**	0.38**	−0.16**	−0.40**	−0.45**		–0.05	−0.10**	−0.11**	0.61**	−0.13**	−0.20**	−0.28**	0.74**
Biology utility	0.39**	0.31**	0.27**	−0.06*	0.41**	0.35**	0.19**	−0.06*		0.61**	0.44**	−0.06*	0.66**	0.37**	0.26**	–0.04
Biology interest	0.28**	0.37**	0.25**	−0.08**	0.24**	0.37**	0.21**	−0.09**	0.62**		0.64**	−0.21**	0.40**	0.50**	0.35**	−0.09**
Biology SC	0.24**	0.29**	0.41**	−0.15**	0.23**	0.29**	0.36**	−0.09**	0.48**	0.66**		−0.27**	0.35**	0.37**	0.55**	−0.09**
Biology cost	−0.09**	−0.12**	−0.10**	0.48**	–0.03	–0.05	–0.03	0.45**	−0.14**	−0.30**	−0.30**		–0.03	−0.06*	−0.11**	0.66**
Physics utility	0.32**	0.25**	0.25**	0.01	0.50**	0.42**	0.31**	−0.11**	0.62**	0.42**	0.38**	–0.06		0.60**	0.50**	−0.16**
Physics interest	0.17**	0.31**	0.22**	–0.00	0.32**	0.53**	0.41**	−0.21**	0.37**	0.53**	0.38**	−0.11**	0.61**		0.70**	−0.28**
Physics SC	0.14**	0.19**	0.35**	–0.05	0.31**	0.41**	0.54**	−0.23**	0.31**	0.35**	0.52**	−0.09**	0.53**	0.68**		−0.35**
Physics cost	0.05	–0.04	0.00	0.39**	−0.08**	−0.14**	−0.12**	0.55**	–0.03	−0.10**	−0.11**	0.60**	−0.13**	−0.31**	−0.31**	
Longitudinal corr.																
Finnish utility	0.44**	0.33**	0.22**	−0.13**	0.14**	0.10**	0.00	0.04	0.19**	0.15**	0.11**	–0.06	0.12**	0.06	0.04	0.01
Finnish interest	0.27**	0.50**	0.33**	−0.17**	0.09*	0.11**	0.05	0.02	0.14**	0.22**	0.14**	–0.07	0.12**	0.15**	0.14**	–0.01
Finnish SC	0.20**	0.37**	0.49**	−0.25**	0.16**	0.19**	0.22**	−0.08*	0.12**	0.19**	0.24**	−0.14**	0.12**	0.14**	0.19**	–0.07
Finnish cost	–0.06	−0.15**	−0.22**	0.37**	–0.02	0.01	–0.06	0.15**	–0.01	0.02	–0.02	0.18**	0.09*	0.06	–0.02	0.12**
Math utility	0.20**	0.14**	0.09*	–0.04	0.39**	0.32**	0.25**	−0.15**	0.19**	0.15**	0.08*	–0.03	0.23**	0.19**	0.14**	–0.03
Math interest	0.14**	0.19**	0.16**	–0.02	0.35**	0.57**	0.52**	−0.29**	0.23**	0.26**	0.20**	–0.04	0.30**	0.38**	0.33**	–0.06
Math SC	0.08*	0.10**	0.22**	–0.05	0.31**	0.51**	0.67**	−0.39**	0.13**	0.22**	0.25**	−0.08*	0.24**	0.35**	0.39**	−0.11**
Math cost	–0.02	–0.06	−0.08*	0.17**	−0.16**	−0.32**	−0.38**	0.42**	−0.10**	−0.09*	–0.07	0.14**	−0.10**	−0.16**	−0.21**	0.21**
Biology utility	0.20**	0.19**	0.10**	–0.02	0.17**	0.23**	0.13**	–0.01	0.44**	0.41**	0.30**	–0.04	0.35**	0.24**	0.21**	–0.01
Biology interest	0.14**	0.22**	0.12**	–0.01	0.16**	0.25**	0.17**	–0.04	0.41**	0.57**	0.43**	−0.18**	0.28**	0.27**	0.25**	–0.07
Biology SC	0.10**	0.17**	0.18**	–0.02	0.13**	0.22**	0.29**	−0.08*	0.31**	0.47**	0.53**	−0.19**	0.25**	0.26**	0.30**	–0.03
Biology cost	–0.05	−0.11**	−0.09*	0.23**	–0.02	–0.06	−0.07*	0.23**	−0.10**	−0.16**	−0.19**	0.35**	–0.00	–0.04	–0.06	0.23**
Physics utility	0.18**	0.15**	0.07	0.03	0.30**	0.31**	0.23**	−0.14**	0.32**	0.29**	0.21**	–0.04	0.47**	0.37**	0.31**	–0.07
Physics interest	0.14**	0.18**	0.15**	0.04	0.26**	0.39**	0.36**	−0.19**	0.27**	0.29**	0.26**	–0.05	0.43**	0.48**	0.46**	−0.13**
Physics SC	0.09*	0.15**	0.22**	–0.02	0.30**	0.40**	0.49**	−0.25**	0.20**	0.28**	0.33**	−0.08*	0.36**	0.42**	0.50**	−0.16**
Physics cost	–0.01	–0.06	−0.08*	0.19**	−0.13**	−0.20**	−0.22**	0.34**	−0.08*	–0.05	−0.08*	0.21**	−0.11**	−0.15**	−0.22**	0.30**
Grade 7																
Mean	5.46	3.93	5.09	3.53	5.82	4.20	4.83	4.19	4.78	4.18	4.74	3.74	4.69	4.01	4.37	4.13
SD	1.52	1.72	1.32	1.83	1.39	1.88	1.67	1.96	1.52	1.85	1.42	1.78	1.64	1.94	1.57	1.82
*N*	1,278	1,251	1,274	1,239	1,265	1,250	1,255	1,221	1,268	1,250	1,252	1,208	1,244	1,223	1,214	1,195
Grade 8																
Mean	5.52	4.24	5.19	3.65	5.73	4.39	4.74	4.32	4.81	4.29	4.82	3.87	4.75	4.05	4.37	4.41
SD	1.58	1.83	1.43	1.96	1.50	1.98	1.77	2.03	1.62	1.90	1.50	1.87	1.80	2.05	1.76	1.93
*N*	1,157	1,143	1,150	1,119	1,151	1,140	1,148	1,118	1,148	1,139	1,145	1,112	1,149	1,139	1,141	1,116
Range	1–7	1–7	1–7	1–7	1–7	1–7	1–7	1–7	1–7	1–7	1–7	1–7	1–7	1–7	1–7	1–7
																

Cross-sectional correlations under the diagonal for Grade 7 and above the diagonal for Grade 8; longitudinal correlations are under the cross-sectional estimates. SC, self-concept; GPA, grade point average of the measured subject domains; SD, standard deviation of the estimate. **p < 0.01, *p < 0.05.

**TABLE 2:** Descriptive data of achievement and occupational aspirations.

	*n*	M	SD
GPA 8	1,302	8.14	1.07
GPA 9	1,219	8.19	1.14
	Frequency of named aspirations for *n* = 413 (full sample for open-answered question)		
	***n***	**%**	**Gender ratio per aspiration (female in %)**
Health STEM	114	27.5	23.5
Other STEM	43	10.4	2.9
STEM (combined)	155	35.5	26.4
non-STEM	257	62.1	37.8

M, mean; SD, standard deviation of the mean estimate.

**TABLE 3:** Model fit criteria of the one- to five-class solutions at T1 (Grade 7) and at T2 (Grade 8).

Model	No of profiles	#fp	LL	Scaling	AIC	BIC	aBIC	Entropy	Smallest likelihood (profile)	Size of smallest profile	LRT test
Grade 7 profile enumeration (*N* = 1,309)	1	32	−38,321.653	0.9010	76,707.306	76,872.971	76,771.322	1			
	2	49	−36,574.940	1.1353	73,247.881	73,501.555	73,345.905	0.857	0.947 (1)	40.9%	0.0000
	3	66	−36,136.697	1.2788	72,405.393	72,747.076	72,537.426	0.812	0.905 (2)	19.8%	0.0008
	**4**	**83**	**−35,889.798**	**1.3809**	**71,945.596**	**72,375.289**	**72,111.637**	**0.813**	**0.870 (1)**	**16.0%**	**0.1330**
	5	100	−35,621.929	1.5753	71,443.858	71,961.560	71,643.907	0.810	0.859 (1)	8.5%	0.5530
Grade 8 profile enumeration (*N* = 1,176)	1	32	−36,221.915	0.8680	72,507.831	72,670.067	72,568.423	1			
	2	49	−34,548.418	1.1735	69,194.836	69,443.259	69,287.618	0.867	0.951	38.4%	0.0000
	3	66	−33,998.197	1.2870	68,128.394	68,463.005	68,253.366	0.830	0.896	34.1%	0.0036
	**4**	**83**	**−33,596.625**	**1.3504**	**67,359.251**	**67,780.050**	**67,516.413**	**0.834**	**0.880 (1)**	**20.5%**	**0.0186**
	5	100	−33,200.750	1.3669	66,601.500	67,108.487	66,790.852	0.866	0.889	2.9%	0.0204

#fp, free parameters; LL, log likelihood; Scaling, log L (MLR corr. factor); aBIC, sample size adjusted BIC, LRT test, LRT test for k vs. k-1 profile. Bold values refer to the chosen profile solution.

#### 3.2.3 Achievement data

Students’ achievement data in Finnish language, math, biology, and physics were retrieved from the registry of the Finnish National Agency for Education. The achievement data were further used as a mean sum score of general GPA in the analyses because it has been shown that academic performance has high correlations across domains in basic education, meaning that students who perform well in math most often perform well in also language (Kupiainen et al., 2014).

#### 3.2.4 Background information

The background information collected in the questionnaire included gender (0 = girl, 1 = boy) and age (i.e., date of birth).

### 3.3 Analytical strategy

In preliminary analysis the descriptive data and correlations of the study variables were examined (see Table 1). Latent profile analysis offer a way to detect different motivation patterns of self-concept and positive and negative task values that might vary across multiple domains. The strength of this analysis is to reveal subgroups in student population that would remain hidden in the average mean level scrutiny. To examine RQ1, the LPAs were conducted separately for each time point including task values across Finnish language, math, and physics. The established profile solutions were based on the akaike information criterion (AIC), the bayesian information criterion (BIC), the sample-size-adjusted Bayesian information criterion (aBIC), and the adjusted Lo-Mendell-Rubin likelihood ratio test (LMR LRT) to examine the difference in the model fit (Nylund et al., 2007). A model with lower AIC, BIC, and aBIC values was considered the best fit to the data. Classification quality was considered in terms of entropy and average class probability for the most likely class membership. In addition, the theoretical interpretation of the profiles and the number of cases in the profiles were considered in the model selection where profiles *n* > 5% of the sample are typically not considered as relevant subgroups (see the guidelines provided by Marsh et al., 2009) (see Table 3 for model fit criteria in LPA).

To examine RQ2, stability and change in the students’ latent profile membership were examined with latent transition analysis (LTA) (Asparouhov and Muthen, 2014). This was done by first testing measurement invariance with longitudinal constraints across the measurement points including profile similarity (Model 1–5), and second, by estimating the transition with saved class probabilities (Model 6). The advantage of using LTA in estimating the transition is that it uses the probability in estimation; thus, instead of fixed groups of students, the uncertainty of the profile membership in each time point is considered (Asparouhov and Muthen, 2014).

After the transition analysis, the auxiliary models were estimated using a manual R3STEP approach (Asparouhov and Muthen, 2014), which produced outputs that could be interpreted as multinomial logistic regression. We first tested gender moderation (Model 7 with free relations and Model 8 with equal relations) in order to later examine gendered effects reliably, and then estimated how gender predicts profile membership to examine RQ5. After this, we examined RQ3 by predicting students’ academic achievement by their GPA in matching domains a year later (Model 9a and 9b).

Finally, to examine RQ4, students’ STEM aspirations in Grade 9 were predicted with Grade 8 profiles (Model 10a and 10b); these analyses were also performed separately for aspirations coded as health science STEM (Model 11a and 11b) and math and natural science STEM (Model 12a and 12b).

All the models were first estimated with direct effects without gender as a covariate (Model a), and then gender was added to the models as a covariate to estimate the gendered effect in order to answer RQ5 (Model b). All models were estimated using Mplus 8.6 (Muthen and Muthen, 2018) and are presented in Table 4.

**TABLE 4:** Model fit criteria for the latent transition analyses.

	#fp	LL	Scaling	AIC	BIC	ABIC
Longitudinal latent profile analysis						
Model 1. Configural similarity	166	−69,486.423	1.3658	139,304.847	140,207.814	139,680.452
Model 2. Configural with residual correlations	278	−67,820.254	3.2116	136,196.507	137,708.705	136,825.532
Model 3. Dispersion similarity (fixed variances)	214	−67,841.538	1.7089	136,111.075	137,275.141	136,595.289
Model 4. Structural similarity (fixed means)	150	−67,742.317	1.4141	135,784.634	136,600.568	136,124.036
Model 5. Distributional similarity (fixed class probabilities)	147	−67,745.898	1.4185	135,785.795	136,585.410	136,118.409
Model 6. Latent transition analysis	15	−3,523.875	0.8668	7,077.749	7,159.343	7,111.689
Predictive similarity						
Model 7. Free relations with predictor (Gender)	21	−3,354.448	0.9096	6,750.895	6,864.063	6,797.350
Model 8. Equal relations with predictor (Gender)	18	−3,355.527	0.8969	6,747.054	6,844.056	6,786.873
Explanatory similarity						
Model 9a. Relations with GPA (without covariate)	25	−7,267.335	0.8565	14,584.670	14,720.659	14,641.237
Model 9b. Relations with GPA (with covariate)	28	−6,031.242	0.9284	12,118.485	12,269.375	12,180.424
Model 10a. Relations with combined STEM (without covariate)	20	−3,799.035	0.8626	7,638.070	7,746.861	7,683.324
Model 10b. Relations with combined STEM (with covariate)	21	−3,641.385	0.8702	7,324.770	7,437.938	7,371.224
Model 11a. Relations with health science STEM (without covariate)	20	−3,770.207	0.8869	7,580.414	7,689.205	7,625.668
Model 11b. Relations with health science STEM (with covariate)	21	−3,595.942	0.8875	7,233.885	7,347.053	7,280.340
Model 12a. Relations with math and natural science STEM (without covariate)	20	−3,616.361	1.0344	7,272.721	7,381.512	7,317.975
Model 12b. Relations with math and natural science STEM (with covariate)	21	−3,447.148	1.0472	6,936.296	7,049.464	6,982.751

#fp, free parameters; LL, log likelihood; Scaling, log L (MLR corr. factor); ABIC, sample size adjusted BIC.

This project used a snowball strategy to recruit the sample; new students were included each year to compensate for the loss of previous-wave students. Of the *N* = 1,702 students, 768 were present in both the Grade 7 and 8 measurement points. Little’s MCAR test showed that data was not missing completely at random (Chi-Square = 4,458.804 DF = 4,262, *p* = 0.018). Therefore, all models were estimated using the robust maximum likelihood estimator (MLR) with full information maximum likelihood (FIML) to handle the missing data; all the available information was used to maximize the sample size and achieve reasonable generalizability.

## 4 Results

### 4.1 Motivation profiles

**FIGURE 1:**
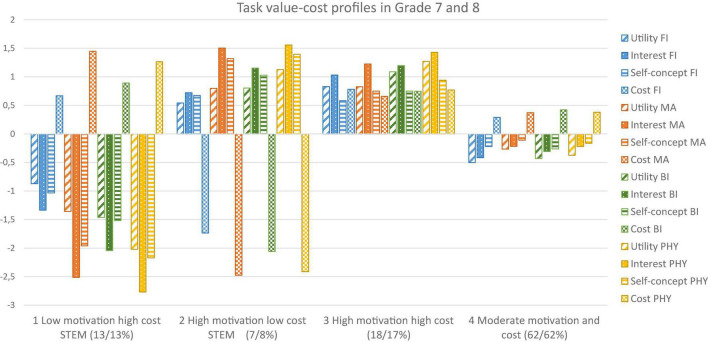
Mean levels of students’ task value-cost profiles in Grade 7 and 8. Means in the figure are centered by the mean of model estimated group means; Proportion of the profiles indicate Grade 7/Grade 8 percentages; FI, Finnish language; MA, Mathematics; BI, Biology; PHY, Physics.

Four similar task value profiles were identified in Grade 7 and 8 (see Figure 1 for centered mean differences). *Low motivation high cost STEM* (13% t1; 13% t2) showed the lowest utility value, interest, and self-concept with the highest cost across domains, and notably low interest and high cost in math and physics. *High motivation low cost STEM* (7% t1; 8% t2) was the smallest profile in both time points and showed the highest utility value, interest, and self-concept with the lowest cost across domains, and again particularly in math and physics. *High motivation high cost* (18% t1; 17% t2) also showed high utility value, interest, and self-concept, accompanied with relatively high cost across domains. *Moderate motivation and cost* (62% t1; 62% t2) was the largest profile and showed moderate task values and cost across domains. The last two profiles showed no clear differences between domains.

### 4.2 Stability of the profile memberships and transition patterns

**FIGURE 2:**
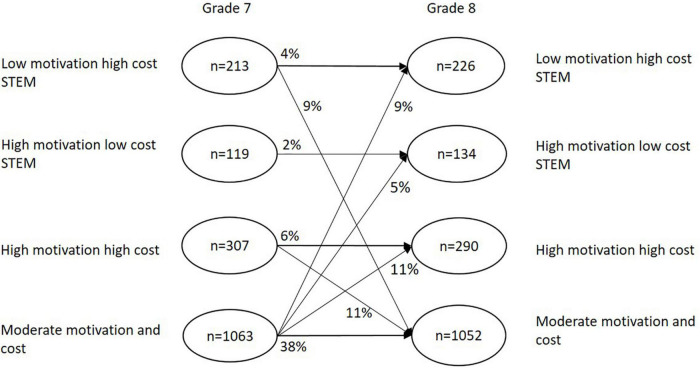
Latent transition patterns with *N* = 1,702 cases. Only the changes that occurred in more than 4% of the total sample (*N* = 1,702) with FIML estimation are depicted. All other changes are reported in Supplementary Table 1. The numbers in the circles refer to the final class proportions for each latent class variable based on their most likely class membership. The numbers on the arrows refer to transition probabilities for the latent class changes based on the estimated model.

Latent transition analysis revealed that students were most likely to move to a *Moderate motivation and cost* profile or remain in their original profile from time 1 to time 2. *Moderate motivation and cost* was the largest and most stable profile across Grade 7 and 8 (transition probability 0.65). The *High motivation low cost STEM* profile was the least stable (transition probabilities 0.26), and *Low motivation high cost STEM* and *High motivation high cost* were slightly more stable profiles (transition probabilities 0.34 and 0.32, respectively) (see Table 5 for details). The transition patterns (Figure 2) indicated that the most frequent transitions across profiles were between *High motivation high cost* and *Moderate motivation and cost* (P3→P4 10.9% and P4→P3 10.5%) as well as between *High motivation low cost STEM* and *Moderate motivation and cost* (P1→P4 8.5% and P4→P1 8.9%). Students that were identified in the smallest and least stable profile *High motivation low cost STEM* were more likely to transition to the *Moderate motivation and cost* profile (3.9%). The percentages provided in the study represent the proportion of students in the total sample (*N* = 1,702 using FIML; Details are shown in Table 5).

**TABLE 5:** Latent transition probabilities from grade 7 to 8.

	Transition probabilities to grade 8 profiles			
Profiles at grade 7	Low motivation high cost STEM	High motivation low cost STEM	High motivation high cost	Moderate motivation and cost
Low motivation high cost STEM	0.335	0.004	0.000	0.660
High motivation low cost STEM	0.040	0.264	0.121	0.574
High motivation high cost	0.000	0.082	0.323	0.595
Moderate motivation and cost	0.134	0.069	0.152	0.645

### 4.3 Differences in academic achievement

Students’ profile memberships in Grades 7 and 8 predicted their academic achievement a year later; in addition, statistically significant differences in the future achievement of the profiles were found. Academic achievement was lowest in the *Low motivation high cost STEM* profile and highest in the *High motivation low cost STEM* profile. Students’ academic achievement (GPA) in Grade 8 differed between the profiles except between the two high motivation profiles: *High motivation low cost STEM* and *High motivation high cost* (Table 6). Students’ achievement in Grade 9 was statistically significant between all the profiles when gender was not in the model as a covariate. However, when the gendered effect was present in the model, the differences between the profiles became non-significant and more complex: only *Low motivation high cost STEM* and *High motivation high cost* profiles remained statistically different in terms of students’ academic achievement (see Table 6 for details).

**TABLE 6:** Task value-profiles and academic achievement and STEM aspirations.

	P1: Low motivation high cost STEM	P2: High motivation low cost STEM	P3: High motivation high cost overall	P4: Moderate motivation and cost overall
	M [SE]	M [SE]	M [SE]	M [SE]
Grade 8				
Relations with GPA (without covariate)	7.55 [0.11]	8.69^a^ [0.10]	8.54^a^ [0.08]	8.10 [0.04]
Relations with GPA (with covariate)	8.27 [0.06]	8.52^a^ [0.06]	8.47^a^ [0.05]	8.38 [0.04]
Grade 9				
Relations with GPA (without covariate)	7.62 [0.09]	8.94 [0.09]	8.50 [0.08]	8.12 [0.05]
Relations with GPA (with covariate)	8.38^a^ [0.06]	8.50^ab^ [0.06]	8.51^b^ [0.05]	8.43^ab^ [0.04]
Relations with STEM (without covariate)	0.13 [0.05]	0.55^a^ [0.09]	0.52^a^ [0.06]	0.32 [0.03]
Relations with STEM (with covariate)	0.15 [0.05]	0.59^a^ [0.09]	0.55^a^ [0.07]	0.35 [0.04]
Relations with health science STEM (without covariate)	0.14^c^ [0.05]	0.38^ab^ [0.09]	0.42^a^ [0.06]	0.24^bc^ [0.03]
Relations with health science STEM (with covariate)	0.20 [0.05]	0.50^ab^ [0.08]	0.53^a^ [0.06]	0.34^b^ [0.04]
Relations with MPECS STEM (without covariate)	0.04^a^ [0.03]	0.22^b^ [0.08]	0.10^ab^ [0.04]	0.10^b^ [0.02]
Relations with MPECS STEM (with covariate)	0.00^a^ [0.03]	0.15^a^ [0.08]	0.03^a^ [0.03]	0.05^a^ [0.02]

Means sharing the same superscript are not significantly different at *p* < 0.05. Means without the superscript accordingly significantly differ from all other profiles, marginally significant differences at *p* < 0.06 are marked with gray superscript.

### 4.4 Differences in STEM aspirations

Students’ STEM aspirations in Grade 9 differed according to their profile membership in Grade 8. Students in the profiles *High motivation low cost STEM* and *High motivation high cost* did not differ in terms of combined STEM aspirations; in addition, the students in these two profiles were more likely to have STEM aspirations compared to students in the profiles *Low motivation high cost STEM* and *Moderate motivation and cost*. Similarly, students in the profiles *High motivation low cost STEM* and *High motivation high cost* did not differ in terms of health science STEM aspirations (coding: health science STEM vs. others), and they were more likely to have health science STEM aspirations compared to students in the profiles *Low motivation high cost STEM* and marginally significantly to *Moderate motivation and cost* when gender was added to the model. However, the significant difference between the profiles were small *High motivation low cost STEM* profile and the *Moderate motivation and cost* profile were not found in the model without gender. Only marginal profile differences were found in students’ math and natural science STEM aspirations (coding: math and natural science vs. others): in the model without gender as a covariate, the *Low motivation high cost STEM* profile was different from the *High motivation low cost STEM* profile (β = −0.137, SE = 0.071, *p* = 0.053). These differences where not found in the model when gender was added as a covariate (see Table 6 for further details).

### 4.5 Gendered differences in motivational profiles and STEM aspirations

Gendered variations in the profile memberships were found in both time points. In Grade 7, more boys than girls belonged to the *Low motivation high cost STEM* profile and the boys were less likely to belong to the other profiles, namely *High motivation low cost STEM*, *Moderate motivation and cost*, and *High motivation high cost*. In Grade 8, in comparison to girls, boys also belonged to the *Low motivation high cost STEM* profile more often than the *Moderate motivation and cost* profile, while the differences between the other profiles were no longer observed (Table 7). Girls were associated with higher academic achievement in both time points compared to boys. In addition, girls had more combined STEM aspirations and were more likely to report occupational aspirations in health science STEM, while boys were more likely to report occupational aspirations in math and natural science STEM in comparison to girls (Table 8).

**TABLE 7:** Effect of gender on latent profile membership.

	OR	SE	95% CI
Grade 7			
P1 vs. P2	0.37***	0.10	[0.22; 0.62]
P1 vs. P3	0.50**	0.11	[0.32; 0.77]
P1 vs. P4	0.47***	0.09	[0.33; 0.69]
P2 vs. P3	1.34	0.33	[0.83; 2.16]
P2 vs. P4	1.28	0.26	[0.85; 1.92]
P3 vs. P4	0.95	0.15	[0.70; 1.30]
Grade 8			
P1 vs. P2	0.60	0.16	[0.36; 1.02]
P1 vs. P3	0.65	0.15	[0.42; 1.03]
P1 vs. P4	0.54**	0.10	[0.37; 0.78]
P2 vs. P3	1.08	0.27	[0.67; 1.75]
P2 vs. P4	0.89	0.19	[0.59; 1.35]
P3 vs. P4	0.82	0.13	[0.60; 1.13]

*N* = 1,618. 0, girls; 1, boys; OR, odds ratios; SE, standard error; 95% CI, 95% confidence intervals. < 0.05, **p < 0.01, ***p < 0.001.

**TABLE 8:** Gendered effects on achievement and STEM aspirations.

	β	SE	*p*
Grade 8			
Gendered effect on GPA	−0.514	0.057	0.000
Grade 9			
Gendered effect on GPA	−0.444	0.063	0.000
Gendered effect on STEM	−0.097	0.048	0.043
Gendered effect on health science STEM	−0.273	0.040	0.000
Gendered effect on natural science STEM	0.155	0.036	0.000

0 = girls, 1 = boys.

## 5 Discussion

During the middle school years, students’ motivation becomes more differentiated and begins to direct their future occupational aspirations (Gaspard et al., 2017; Guo et al., 2017). Students report diverse expectancies and values: motivational patterns are formed by the intraindividual hierarchies of task values and costs that vary among students and across domains (Gaspard et al., 2019). This study contributes to the expectancy-value literature in several ways: first, by investigating the associations between the positive and negative task values simultaneously across multiple domains using a longitudinal person-oriented approach; second, by investigating the stability of the identified task value-cost profiles over time; third, by examining how the task value-cost profiles are associated with subsequent academic achievement and STEM aspirations; and fourth, by examining the possible gender differences in students’ task value-cost profiles, academic achievement, and STEM aspirations. In addition, this study uses more nuanced categorization to examine students’ STEM aspirations in the fields of math and natural sciences, and health and medical sciences providing relevant information of gendered career aspirations.

### 5.1 Motivation profiles

Four task value-cost profiles were identified in Grades 7 and 8. *Low motivation high cost STEM* (13% t1; 13% t2) showed the lowest task values with the highest cost across all domains, but especially in math and physics. In turn, *High motivation low cost STEM* (7% t1; 8% t2) showed high task values and low cost, especially in math and physics. *High motivation high cost* (18% t1; 17% t2) showed high task values accompanied with relatively high cost across domains. *Moderate motivation and cost* (62% t1; 62% t2) showed moderate task values and cost across domains. The *High motivation low cost STEM* profile was the smallest group, whereas the *Moderate motivation and cost* was clearly the largest profile at both time points.

The results of this study supported earlier findings and confirmed our hypothesis regarding the number of profiles and the task value-cost patterns. Four profiles were identified, which is typical in person-oriented studies using task values (Chow et al., 2012; Guo et al., 2018; Lazarides et al., 2019). The task value and cost patterns also resembled the profiles that have been found in previous studies using the positive and negative aspects of the task values (Lee et al., 2022) and across math and English as the second language (Gaspard et al., 2019). The profiles *High motivation high cost* and *High motivation low cost STEM* confirmed our hypothesis that high motivation patterns would be observed with high and low cost. In addition, *Low motivation high cost STEM* exhibited the expected low motivation pattern. The profiles *High motivation low cost STEM* and *Low motivation high cost STEM* showed patterns of mixed motivation across domains and confirmed our hypothesis (Gaspard et al., 2019; Oppermann et al., 2021). Finally, the *Moderate motivation and cost* profile demonstrated the expected pattern with average task values.

In this study, over half of the students belonged to the *Moderate motivation and cost* profile, which confirms the findings of earlier studies that did not identify clearly differentiated task values and costs among groups of students (Perez et al., 2019; Watt et al., 2019). This finding indicates that the majority of middle school students are somewhat motivated to study, and they have not yet have developed highly distinguished task values in Finnish language, math, biology, and physics; in addition, middle school students feel moderately exhausted by their studies in all domains. This could be considered as a typical student in Middle school. The *High motivation profile with high cost* depict a typical high achieving student, most likely girl, who is highly motivated toward school and is determined to perform well in all domains. This profile could be in risk of studyholism and study burnout. However, two smaller groups of students report high or low positive task values especially in STEM domains depicting two opposite motivation patterns. It seems that STEM domains divide student motivation clearly into two groups where students are either highly motivated in math and physics with no perceived cost or considerably unmotivated in math and physics with high cost.

### 5.2 Stability of the profiles and transitions in profile membership

Latent transition analysis further revealed that *Moderate motivation and cost* was the most stable profile over time; the other profiles showed rather low stability. Previous research that has used LTA to examine patterns of students’ expectancies and values has found moderately stable motivation profiles (e.g., Oppermann et al., 2021), but low stability has also been observed to some extent (Lazarides et al., 2021). However, these studies have only included the positive task values across domains. This study investigated task values and cost simultaneously across several domains, and thus provides new insights by showing that as the variation in the motivation profiles increases it may result in reduced stability over time. Moreover, middle school students undergo major developmental changes (e.g., puberty, adjustment to the school transition from primary to middle school, changes in peer relations), which may affect their academic motivation. Therefore, task motivation might be more prone to changes in middle school when internal and external frames of reference influence the hierarchies of students’ expectancies and values in many subjects (see Marsh, 1990). Especially math physics become increasingly difficult in middle school resulting changes in students’ self-concept, interest and utility values, and emotional cost in these domains when students proceed from grade 7 to 8. This might also explain the low stability in *High motivation low cost STEM* profile. Peer interactions affect students’ self-perception and motivation, and social desirability might influence especially girls’ motivation in math and physics. Additional longitudinal research is required to explore the cross-domain patterns of task values and cost.

### 5.3 Motivation profiles and academic achievement

Students’ profile membership in Grades 7 and 8 predicted their academic achievement a year later, and the profiles differed according to students’ academic achievement. As expected, the high motivation profiles, namely *High motivation low cost STEM* and *High motivation high cost*, were associated with the highest academic achievement, while the *Low motivation high cost STEM* profile was shown to have the lowest academic achievement. *Moderate motivation* profile showed moderate achievement; significantly lower than the two high motivation profiles but higher than the *Low motivation high cost STEM* profile. In Grade 8, no differences in students’ GPA were found between the two high motivation profiles; however, differences were present in Grade 9. Moreover, when gender was included in the model, the differences between the profiles became non-significant and more complex: students in the *Low motivation high cost STEM* profile had a lower GPA compared to students in the *High motivation high cost* profile when students’ gender was taken into account. These findings indicate that the association between student motivation and subsequent academic performance become stronger when students continue to pursue their educational path, and gender may play a role in this relationship by showing more differentiated motivation patterns and less clear achievement gaps between male and female students.

### 5.4 Motivation profiles and STEM aspirations

The results showed that students who reported *High motivation low cost STEM* or *High motivation high cost* and, to some extent, students with a *Moderate motivation and cost* profile had more combined STEM aspirations than students belonging to the *Low motivation high cost STEM* profile. This finding partially confirms our hypothesis that high motivation profiles in math and science and/or high motivation across domains is associated with more STEM aspirations compared to other profiles, an observation that is also in line with existing literature (Chow et al., 2012; Guo et al., 2018; Oppermann et al., 2021). Seems plausible that students in *Low motivation high cost STEM* profile who have low self-concept and hold low interest and utility value in math and physics and simultaneously experience high emotional cost in these domains result having no future career aspirations in STEM. The two high motivation profiles identified in this study did not show any differences in terms of students’ STEM aspirations. Overall, only half of the students provided an answer when asked about a future occupation that they would want to pursue, and the majority of the occupations were coded as non-STEM. Health science STEM occupations were more frequently identified than careers in the math and natural science STEM fields. The low number of STEM aspirations might be the result of the non-significant findings between the profiles; it is possible that the students who indicated high motivation had already clearly established their future outlooks and thus were aware of more STEM occupations than the students who showed low overall motivation toward school.

### 5.5 Gendered motivation and STEM aspirations

This study showed significant gendered variation in the profile memberships at both time points. In Grade 7, male students were more likely to have a *Low motivation high cost STEM* profile and were less likely to belong to the other profiles, namely *High motivation low cost STEM*, *High motivation high cost*, and *Moderate motivation and cost*. In Grade 8, it was also more likely for a male student to report *Low motivation high cost STEM* than *Moderate motivation and cost*. The overrepresentation of boys in the low motivation profile is in line with frequently reported gender differences, as is the overrepresentation of girls in the high motivation profile (Chow et al., 2012; Oppermann et al., 2021). However, in the literature discussing expectancies and values, the majority of studies have reported higher motivation among boys in math and science (Watt, 2016), and this observation was not clearly replicated in this study. Most of the students who named a future occupation were girls. Moreover, the female students named non-STEM occupations more frequently than STEM occupations, and the majority of the STEM occupations were in health science STEM fields. The boys in this study named more math and natural science STEM occupations than the girls. These gendered STEM aspirations are also in line with the findings described in the existing literature (Dicke et al., 2019; Toh and Watt, 2022).

### 5.6 Practical implications and interventions

While there is significant awareness of the need to improve girls’ engagement (UNESCO, NSF) in STEM fields, gender biases and stereotypes are still prevalent, creating obstacles to the recruitment and progression of girls in STEM education and careers. Results from intervention studies (e.g., Rosenzweig et al., 2020) have suggested that cost reduction and utility value interventions are both useful tools for improving students’ STEM course performance. However, girls’ academic achievement in middle school does not appear to be related to the limited number of female students pursuing a future in STEM education and careers; instead, a lack of interest in STEM fields and a stronger focus, in particular, on the internal hierarchies of other occupations may explain why girls rarely aspire to physical science occupations. By providing girls more knowledge and hands-on interactive STEM activities, it would be possible to promote girls’ STEM motivation and aspirations (Franz-Odendaal and Marchand, in press) and positive emotions in science class (Itzek-Greulich and Vollmer, 2017). For example, intervention programs which would involve students discussing with role models (e.g., women working in STEM fields) may provide girls better insights into STEM careers and inspire girls to be more engaged in STEM domains (Franz-Odendaal and Marchand, in press). Moreover, previous studies have shown that female students often feel that they do not belong to STEM fields, leading them to pursue other than STEM careers (Aelenei et al., 2020). Interventions targeting sense of belonging and providing students collaborative tasks where they can work together for a common goal may support female students’ interest in STEM fields (Aelenei et al., 2020). Motivation-emotion relationship should be better acknowledged in science education; by modifying teaching methods it may be possible to evoke positive achievement emotions and boost students’ situational motivation in the science learning context (Itzek-Greulich and Vollmer, 2017). The findings of the current study do not show that girls experience more cost in math and physics, rather some girls may experience a cost associated with high motivation across domains. It is important to harness this high motivation and direct it into STEM-related fields; thus, there is a need to design interventions that would compensate for female students’ missed opportunities to engage in science activities (Murphy and Whitelegg, 2006).

## 6 Conclusion

This study identified four profiles among students in middle school: two STEM-oriented profiles, one with high motivation and low cost and the other with low motivation and high cost, especially in math and physics, and two profiles depicting high motivation and cost across domains and moderate motivation and cost across domains. The moderate motivation profile was the largest and most stable profile across both Grades 7 and 8. Gendered variations in the profile memberships and STEM aspirations were also observed: girls were more likely to belong to the high motivation profiles or a moderate motivation profile, while more boys reported having a low motivation and high cost profile. Moreover, girls showed higher academic achievement in comparison to boys and had more life science STEM aspirations; in contrast, boys reported more STEM aspirations in the physical sciences. The results suggest that the majority of middle school students are moderately to highly motivated in various domains; however, some students simultaneously experience a high cost, which may reflect the increase in course difficulty and study-related demands in middle school.

### 6.1 Limitations and further research

Our longitudinal study was conducted with middle school students in Helsinki, Finland and included a relatively large number of participants. However, it should be noted that the participation of the same students varied across the time points. Most of the students recruited in Grade 7 remained in the study in Grade 8; however, in Grade 9, the data collection attrition increased and resulted in limited data on STEM aspirations. Students’ future occupational aspirations were measured with an open-ended question that only yielded 413 answers that were further coded as non-STEM/STEM. The data for this study was collected in 21 middle schools from across the Helsinki metropolitan area and included students from various family backgrounds. However, as population in Finland is rather homogeny regarding race/ethnicity and socioeconomic background, a proper information of the SES was not collected. Further research is required to confirm the validity of the observations and the generalizability of the findings; for example, it would be desirable to extend the focus by including students from different Finnish cities or regions and even other countries. The use of a one-item task value measure in the data collection meant that we could not test the reliability of the scale, and this may weaken the validity of the study. However, we employed LPA to reduce the measurement error. While LPA is a useful means of identifying possible subgroups in the population, there are possible shortcomings related to the person-oriented methodology. We should bear in mind that the results of students’ high/average/low level of task values are always relative to the used sample and cannot be interpret as objective information of student motivation in general. Moreover, these results might be different if the same analyses were conducted using another sample or in other population. In person-oriented techniques, such as LPA, the researcher is responsible for selecting and interpreting the final profile solution. While identifying profiles in the data can appear relatively straightforward, it can be difficult to classify a student in only one profile. In this study, we carefully followed standard guidelines (Asparouhov and Muthen, 2014) when conducting the LTA and confirmed that the results were aligned with the underlying theoretical framework and previous research.

The interaction of individual and contextual factors could be considered in future research. Collecting data on students’ everyday experiences during classes may reveal the immediate interplay between interest and costs which could help researchers to understand the formation of students’ more permanent motivation beliefs toward different domains and future career aspirations. It would also be beneficial to investigate students’ levels of interest and their simultaneous perceptions of cost when engaged in different tasks within a domain (for example, math or science), and how the in-the-moment interplay is related to students’ STEM aspirations. For educators, it would be important to understand the possibilities to influence task motivation in the classroom and inspire students to STEM. Moreover, it would be interesting to consider if friends share similar patterns of interests and costs, and even STEM aspirations. Examining joint motivation patterns within friend groups might reveal synchronous changes in students’ task-values which further contribute to the formation of STEM aspirations as students proceed through the middle school years.

## Data availability statement

The data analyzed in this study is subject to the following licenses/restrictions: The longitudinal dataset contains pseudonymized identifiers of the under aged study participants. At this point of the research project the data cannot be published. Requests to access these datasets should be directed to JV-L, janica.vinni-laakso@helsinki.fi.

## Ethics statement

Ethical review and approval was not required for the study on human participants in accordance with the local legislation and institutional requirements. Written informed consent to participate in this study was provided by the participants’ legal guardian/next of kin.

## Author contributions

JV-L performed the analytic calculations and wrote the manuscript with the help of KU and KS-A. KS-A was responsible for developing the original idea. JV-L and KU planned the modeling technique and the use of previously collected data. KS-A helped to supervise the project. All the authors discussed the results and contributed to the final manuscript.

## Footnotes

1
tilastokeskus.fi

## References

[B1] Aelenei C. Martinot D. Sicard A. Darnon C. (2020). When an academic culture based on self-enhancement values undermines female students’ sense of belonging, self-efficacy, and academic choices. *J. Soc. Psychol.* 160 373–389. 10.1080/00224545.2019.1675576 31600124

[B2] Asparouhov T. Muthen B. (2014). Auxiliary variables in mixture modeling: Three-step approaches using Mplus. *Struct. Equ. Modeling* 21 329–341. 10.1080/10705511.2014.915181

[B3] Barron K. E. Hulleman C. S. (2015). *Expectancy-value-cost model of motivation*, 2nd Edn. Amsterdam: Elsevier, 503–509.

[B4] Bong M. (2001). Between-and within-domain relations of academic motivation among middle and high school students: Self-efficacy, task value, and achievement goals. *J. Educ. Psychol.* 93:23. 10.1037/0022-0663.93.1.23

[B5] Chow A. Salmela-Aro K. (2011). Task-values across subject domains: A gender comparison using a person-centered approach. *Int. J. Behav. Dev.* 35 202–209.

[B6] Chow A. Eccles J. Salmela-Aro K. (2012). Task value profiles across subjects and aspirations to physical and IT-related sciences in the United States and Finland. *Dev. Psychol.* 48 1612–1628. 10.1037/a0030194 23127302

[B7] Conley A. M. (2012). Patterns of motivation beliefs. *J. Educ. Psychol.* 104 32–47.

[B8] Dicke A. L. Safavian N. Eccles J. S. (2019). Traditional gender role beliefs and career attainment in STEM: A gendered story? *Front. Psychol.* 10:1053. 10.3389/fpsyg.2019.01053 31139116 PMC6519300

[B9] Eccles J. (2011). Gendered educational and occupational choices: Applying the Eccles et al. model of achievement-related choices. *Int. J. Behav. Dev.* 35 195–201. 10.1177/0165025411398185

[B10] Eccles J. S. Wigfield A. (2020). From expectancy-value theory to situated expectancy-value theory: A developmental, social cognitive, and sociocultural perspective on motivation. *Contemp. Educ. Psychol.* 61:101859. 10.1016/j.cedpsych.2020.101859

[B11] Eccles J. Adler T. F. Futterman R. Goff S. B. Kaczala C. M. Meece J. L. (1983). “Expectancies, values, and academic behaviors,” in *Achievement and achievement motivation*, ed. Spence J. T. (San Francisco, CA: W. H. Freeman), 75–146.

[B12] Eccles J. Wigfield A. Harold R. D. Blumenfeld P. (1993). Age and gender differences in children’s self-and task perceptions during elementary school. *Child Dev.* 64 830–847. 10.1111/j.1467-8624.1993.tb02946.x 8339698

[B13] Flake J. K. Barron K. E. Hulleman C. McCoach B. D. Welsh M. E. (2015). Measuring cost: The forgotten component of expectancy-value theory. *Contemp. Educ. Psychol.* 41 232–244. 10.1016/j.cedpsych.2015.03.002

[B14] Franz-Odendaal T. Marchand S. (in press). Girls get wise–a programming model for engaging girls+ in STEM. *Front. Psychol.* 10.3389/fpsyg.2022.924943 PMC9807030 36600699

[B15] Fryer L. K. Ainley M. (2019). Supporting interest in a study domain: A longitudinal test of the interplay between interest, utility-value, and competence beliefs. *Learn. Instr.* 60 252–262. 10.1016/j.learninstruc.2017.11.002

[B16] Gaspard H. Dicke A. Flunger B. Schreier B. Häfner I. Trautwein U. (2015). More value through greater differentiation: Gender differences in value beliefs about math. *J. Educ. Psychol.* 107 663–677. 10.1037/edu0000003

[B17] Gaspard H. Häfner I. Parrisius C. Trautwein U. Nagengast B. (2017). Assessing task values in five subjects during secondary school: Measurement structure and mean level differences across grade level, gender, and academic subject. *Contemp. Educ. Psychol.* 48 67–84. 10.1016/j.cedpsych.2016.09.003

[B18] Gaspard H. Wille E. Wormington S. V. Hulleman C. S. (2019). How are upper secondary school students’ expectancy-value profiles associated with achievement and university STEM major? A cross-domain comparison. *Contemp. Educ. Psychol.* 58 149–162.

[B19] Guo J. Marsh H. W. Parker P. D. Morin A. J. Dicke T. (2017). Extending expectancy-value theory predictions of achievement and aspirations in science: Dimensional comparison processes and expectancy-by-value interactions. *Learn. Instr.* 49 81–91. 10.1016/j.learninstruc.2016.12.007

[B20] Guo J. Parker P. D. Marsh H. W. Morin A. J. S. (2015). Achievement, motivation, and educational choices: A longitudinal study of expectancy and value using a multiplicative perspective. *Dev. Psychol.* 51 1163–1176. 10.1037/a0039440 26053150

[B21] Guo J. Wang M.-T. Ketonen E. E. Eccles J. S. Salmela-Aro K. (2018). Joint trajectories of task value in multiple subject domains: From both variable- and pattern-centered perspectives. *Contemp. Educ. Psychol.* 55 139–154. 10.1016/j.cedpsych.2018.10.004

[B22] Hodis F. A. Hodis G. M. (2020). Patterns of motivation and communication in learning environments: A latent profile analysis. *Soc. Psychol. Educ.* 23 1659–1685. 10.1007/s11218-020-09599-3

[B23] Homer M. Ryder J. Banner I. (2014). Measuring determinants of post-compulsory participation in science: A comparative study using national data. *Br. Educ. Res. J.* 40 610–636. 10.1002/berj.3106

[B24] Itzek-Greulich H. Vollmer C. (2017). Emotional and motivational outcomes of lab work in the secondary intermediate track: The contribution of a science center outreach lab. *J. Res. Sci. Teach.* 54 3–28.

[B25] Jacobs J. E. Lanza S. Osgood D. W. Eccles J. S. Wigfield A. (2002). Changes in children’s self-competence and values: Gender and domain differences across grades one through twelve. *Child Dev.* 73 509–527. 10.1111/1467-8624.00421 11949906

[B26] Jiang Y. Rosenzweig E. Q. (2021). Using cost to improve predictions of adolescent students’ future choice intentions, avoidance intentions, and course grades in mathematics and English. *Learn. Individ. Dif.* 86:101978. 10.1016/j.lindif.2021.101978

[B27] Jiang S. Simpkins S. D. Eccles J. S. (2020). Individuals’ math and science motivation and their subsequent STEM choices and achievement in high school and college: A longitudinal study of gender and college generation status differences. *Dev. Psychol.* 56:2137. 10.1037/dev0001110 32915052

[B28] Jiang Y. Rosenzweig E. Q. Gaspard H. (2018). An expectancy-value-cost approach in predicting adolescent students’ academic motivation and achievement. *Contemp. Educ. Psychol.* 54 139–152. 10.1016/j.cedpsych.2018.06.005

[B29] Kang J. Hense J. Scheersoi A. Keinonen T. (2019). Gender study on the relationships between science interest and future career perspectives. *Int. J. Sci. Educ.* 41 80–101. 10.1080/09500693.2018.1534021

[B30] Kupiainen S. Vainikainen M.-P. Marjanen J. Hautamäki J. (2014). The role of time on task in computer-based low-stakes assessment of cross-curricular skills. *J. Educ. Psychol.* 106 627–638. 10.1037/a0035507

[B31] Lazarides R. Dicke A.-L. Rubach C. Oppermann E. Eccles J. S. (2021). Motivational profiles across domains and academic choices within Eccles et al.’s situated expectancy–value theoretical framework. *Am. Psychol. Assoc.* 57 1893–1909. 10.1037/dev0001250 34914452

[B32] Lazarides R. Dietrich J. Taskinen P. H. (2019). Stability and change in students’ motivational profiles in mathematics classrooms: The role of perceived teaching. *Teach. Teach. Educ.* 79 164–175. 10.1016/j.tate.2018.12.016

[B33] Lee S. Y. Friedman S. Christiaans E. Robinson K. A. (2022). Valuable but costly? University students’ expectancy-value-cost profiles in introductory chemistry courses. *Contemp. Educ. Psychol.* 69:102056. 10.1016/j.cedpsych.2022.102056

[B34] Marsh H. W. (1990). The influences of internal and external frames of reference on the formation of English and Math self-concepts. *J. Educ. Psychol.* 82 107–116.

[B35] Marsh H. W. Lüdtke O. Trautwein U. Morin A. J. S. (2009). Classical latent profile analysis of academic self-concept dimensions: Synergy of person- and variable-centered approaches to theoretical models of self-concept. *Struct. Equ. Model.* 16 191–225.

[B36] Martin M. O. Mullis I. V. S. Foy P. Hooper M. (2016). *TIMSS 2015 international results in science.* Boston, MA: TIMSS & PIRLS International Study Center, Boston College.

[B37] Murphy P. Whitelegg E. (2006). Girls and physics: Continuing barriers to ‘belonging.’. *J. Curric.* 17 281–305. 10.1080/09585170600909753

[B38] Muthen L. K. Muthen B. O. (2018). *Mplus user’s guide*, 8th Edn. Los Angeles, CA: Muthén & Muthén. 10.1016/j.lindif.2017.11.017

[B39] Nagy G. Garrett J. Trautwein U. Cortina K. S. Baumert J. Eccles J. S. (2008). “Gendered high school course selection as a precursor of gendered careers: The mediating role of self-concept and intrinsic value,” in *Gender and occupational outcomes: Longitudinal assessments of individual, social, and cultural influences*, Eds Watt H. M. G. Eccles J. S. (Washington, DC: American Psychological Association), 115–143. 10.1037/11706-004

[B40] National Science Foundation [NSF] (2017). *Women, minorities, and persons with disabilities in science and engineering: 2017.* Arlington, TX: National Center for Science and Engineering Statistics.

[B41] Nylund K. L. Asparouhov T. Muthen B. (2007). Deciding on the number of classes in latent class analysis and growth mixture modeling: A monte carlo simulation study. *Struct. Equ. Model.* 14 535–569. 10.1080/10705511.2014.882690 24729675 PMC3979564

[B42] OECD (2016). *PISA 2015 results (volume I): Excellence and equity in education, PISA.* Paris: OECD Publishing.

[B43] Oppermann E. Vinni-Laakso J. Juuti K. Loukomies A. Salmela-Aro K. (2021). Elementary school students’ motivational profiles across Finnish language, mathematics and science: Longitudinal trajectories, gender differences and STEM aspirations. *Contemp. Educ. Psychol.* 64:101927. 10.1016/j.cedpsych.2020.101927

[B44] Perez T. Cromley J. G. Kaplan A. (2014). The role of identity development, values, and costs in college STEM retention. *J. Educ. Psychol.* 106 315–329. 10.1037/a0034027

[B45] Perez T. Wormington S. V. Barger, Schwartz-Bloom R. D. Lee Y. K. Linnenbrink-Garcia L. (2019). Science expectancy, value, and cost profiles and their proximal and distal relations to undergraduate science, technology, engineering, and math persistence. *Sci. Educ.* 103 264–286. 10.1002/sce.21490 31186590 PMC6558974

[B46] Potvin P. Hasni A. (2014). Interest, motivation and attitude towards science and technology at K-12 levels: A systematic review of 12 years of educational research. *Stud. Sci. Educ.* 50 85–129. 10.1080/03057267.2014.881626

[B47] Rosenzweig E. Q. Wigfield A. Hulleman C. S. (2020). More useful or not so bad? Examining the effects of utility value and cost reduction interventions in college physics. *J. Educ. Psychol.* 112 166–182. 10.1037/edu0000370

[B48] Ryan R. M. Deci E. L. (2000). Intrinsic and extrinsic motivations: Classic definitions and new directions. *Contemp. Educ. Psychol.* 25 54–67. 10.1006/ceps.1999.1020 10620381

[B49] Salmela-Aro K. (2017). Dark and bright sides of thriving–school burnout and engagement in the Finnish context. *Eur. J. Dev. Psychol.* 14 337–349. 10.1080/17405629.2016.1207517

[B50] Suomen Virallinen Tilasto [SVT] (2022). *Väestörakenne [verkkojulkaisu].* Helsinki: Tilastokeskus.

[B51] Toh L. Watt H. M. G. (2022). How do adolescent mathematical self-concept and values explain attainment of different kinds of STEM degrees in adulthood? *Contemp. Educ. Psychol.* 69:102057. 10.1016/j.cedpsych.2022.102057

[B52] Tuominen H. Juntunen H. Niemivirta M. (2020). Striving for success but at what cost? Subject-specific achievement goal orientation profiles, perceived cost, and academic well-being. *Front. Psychol.* 11:557445. 10.3389/fpsyg.2020.557445 33117226 PMC7550833

[B53] Tytler R. (2014). “Attitudes, identity, and aspirations toward science,” in *Handbook of research on science education*, eds Lederman N. G. Abell S. K. (New York, NY: Routledge), 82–103. 10.1097/ACM.0000000000002006

[B54] UNESCO (2020). *Boosting gender equality in science and technology. A challenge for TVET programmes and careers.* Paris: United Nations Educational, Scientific and Cultural Organization

[B55] Viljaranta J. Aunola K. Hirvonen R. (2016). Motivation and academic performance among first-graders: A person-oriented approach. *Learn. Individ. Differ.* 49 366–372.

[B56] Vinni-Laakso J. Guo J. Juuti K. Loukomies A. Lavonen J. Salmela-Aro K. (2019). The relations of science task values, self-concept of ability, and STEM aspirations among Finnish students from first to second grade. *Front. Psychol.* 10:1449. 10.3389/fpsyg.2019.01449 31312153 PMC6614377

[B57] Wang M. T. Degol J. (2013). Motivational pathways to STEM career choices: Using expectancy–Value perspective to understand individual and gender differences in STEM fields. *Dev. Rev.* 33 304–340. 10.1016/j.dr.2013.08.001 24298199 PMC3843492

[B58] Wang M.-T. Degol J. L. (2017). Gender gap in science, technology, engineering, and mathematics (STEM): Current knowledge, implications for practice, policy, and future directions. *Educ. Psychol. Rev.* 29 119–140. 10.1007/s10648-015-9355-x 28458499 PMC5404748

[B59] Watt H. (2016). “Gender and motivation,” in *Handbook of motivation at school*, 2nd Edn. eds Wentzel K. R. Miele D. B. (New York, NY: Routledge), 320–339.

[B60] Watt H. M. G. Bucich M. Dacosta L. (2019). Adolescents’ motivational profiles in mathematics and science: Associations with achievement striving, career aspirations and psychological wellbeing. *Front. Psychol.* 10:990. 10.3389/fpsyg.2019.00990 31316409 PMC6610331

[B61] Watt H. M. Shapka J. D. Morris Z. A. Durik A. M. Keating D. P. Eccles J. S. (2012). Gendered motivational processes affecting high school mathematics participation, educational aspirations, and career plans: A comparison of samples from Australia, Canada, and the United States. *Dev. Psychol.* 48 1594–1611. 10.1037/a0027838 22468566

[B62] Wigfield A. Cambria J. (2010). Students’ achievement values, goal orientations, and interest: Definitions, development, and relations to achievement outcomes. *Dev. Rev.* 30 1–35. 10.1016/j.dr.2009.12.001

[B63] Wigfield A. Eccales J. S. (2020). 35 years of research on students’ subjective task values and motivation: A look back and a look forward. *Adv. Motiv. Sci.* 7 161–198. 10.1016/bs.adms.2019.05.002

[B64] Wigfield A. Eccles J. S. Yoon K. S. Harold R. D. Arbreton A. J. Freedman-Doan C. (1997). Change in children’s competence beliefs and subjective task values across the elementary school years: A 3-year study. *J. Educ. Psychol.* 89:451. 10.1037/0022-0663.89.3.451

[B65] Wille E. Stoll G. Gfrörer T. Cambria J. Nagengast B. Trautwein U. (2020). It takes two: Expectancy-value constructs and vocational interests jointly predict STEM major choices. *Contemp. Educ. Psychol.* 61:101858. 10.1016/j.cedpsych.2020.101858

