# Supplementary material for: Associations between adolescent students’ multiple domain task value-cost profiles and STEM aspirations
Source: Front Psychol. 2022 Dec 22;13:951309. doi: 10.3389/fpsyg.2022.951309 (PMC9815538; doi:10.3389/fpsyg.2022.951309)
Supplement: Supplementary file 1 [file Table_1.docx]

Supplementary material

| Table S1. Transition patterns from Grade 7 to 8 | | | |
| --- | --- | --- | --- |
| Grade 7 | Grade 8 | N | % of the sample |
| 1 | 1 | 68 | 4.0% |
| 1 | 2 | 0 | 0 % |
| 1 | 3 | 0 | 0 % |
| 1 | 4 | 145 | 8.5% |
| 2 | 1 | 6 | <1% |
| 2 | 2 | 29 | 1.7% |
| 2 | 3 | 17 | <1% |
| 2 | 4 | 67 | 3.9% |
| 3 | 1 | 0 | 0 % |
| 3 | 2 | 27 | 1.6% |
| 3 | 3 | 94 | 5.5% |
| 3 | 4 | 186 | 10.9% |
| 4 | 1 | 152 | 8.9% |
| 4 | 2 | 78 | 4.5% |
| 4 | 3 | 179 | 10.5% |
| 4 | 4 | 654 | 38.4% |
| *Note:* N=1702. | |  |  |

| Table S2. STEM occupations and number of most frequent nominations (N=155) | | | |
| --- | --- | --- | --- |
| **Health Science STEM** | N | **Math and natural science STEM** | N |
| Crisis doctor in disaster areas |  | Architect | 11 |
| Dentist | 7 | Astronaut |  |
| Doctor | 52 | Astronomer |  |
| Doctor for athletes |  | Car mechanic |  |
| Doctor specialized |  | Coder |  |
| Doctor specialized in dermatology | | Electrician | 3 |
| Medical |  | Engineer | 10 |
| Medicine |  | Engineer in mechanics |  |
| Midwife |  | Game designer |  |
| Neurologist |  | ICT / computer support person |  |
| Neurosurgeon |  | Information and communication technologies | |
| Nurse | 9 | Machining |  |
| Nutritionist |  | Marine biologist |  |
| Pediatrician |  | Mechanic |  |
| Pharmacist |  | Physicist |  |
| Physiotherapist | 5 | Programmer | 3 |
| Psychologist | 8 | Reseacher |  |
| Psychiatrist |  | Something related to computers |  |
| Something related to medical |  | Something related to technology/mechanics |  |
| Speech therapist | 4 | Traffic planner |  |
| Surgeon | 8 | Web designer / Program designer |  |
| Therapist |  |  |  |
| Veterinarian | 9 |  |  |
| Work in care sector |  |  |  |

| Table 3S. Non-STEM occupations (N=257) |  |
| --- | --- |
| **non-STEM occupations** |  |
| A diplomat |  |
| a minister |  |
| Accountant |  |
| Actor | * |
| Anthropologist |  |
| Archaeologist |  |
| Artist |  |
| Athlete |  |
| Author |  |
| Banker |  |
| Beautician |  |
| Blogger |  |
| Business |  |
| Business man |  |
| CEO |  |
| Chef |  |
| Choreographer |  |
| Cleaner |  |
| Coach |  |
| Construction worker |  |
| Copy editor |  |
| Customer service |  |
| Dancer |  |
| Designer / artist |  |
| Designing visuals: pages, logos |  |
| Detective |  |
| Director |  |
| Director in theater or movies |  |
| DJ |  |
| Drive a car |  |
| Economist |  |
| Entrepreneur | * |
| Fashion designer |  |
| Fighter pilot |  |
| Film industry |  |
| Flight attendant |  |
| Florist |  |
| Gamer |  |
| Graphic designer |  |
| Graphic designer for video games |  |
| Historian |  |
| Hockey player |  |
| Human rights lawyer |  |
| In the army |  |
| Interior designer |  |
| Interpreter |  |
| Investment Banker |  |
| Journalist | * |
| Judge |  |
| Lawyer | ** |
| Leader or politician |  |
| Librarian |  |
| Make up artist |  |
| Management |  |
| Mangaka |  |
| Marketing |  |
| Marketing manager |  |
| Model |  |
| Music producer |  |
| Music related work |  |
| Music therapist |  |
| Musician |  |
| Opera singer |  |
| Paratrooper |  |
| pastry cook |  |
| Performance artist |  |
| Personal Trainer |  |
| photographer |  |
| Physical education instructor |  |
| Pianist |  |
| Pilot |  |
| Police officer |  |
| PR agent |  |
| Producer |  |
| Professional Athlete |  |
| Psychologist | ** |
| Real estate mogul |  |
| Sales man |  |
| Sea captain |  |
| Security guy in a bank |  |
| Soccer player |  |
| Social scientist |  |
| Social worker |  |
| Solder |  |
| Solo violinist |  |
| Something creative |  |
| Something creative among people |  |
| Something in game industry |  |
| Something related to animal rights |  |
| Something related to animals |  |
| Something related to art |  |
| Something related to culture |  |
| Something related to fashion |  |
| Something related to law |  |
| Something related to literature |  |
| Something related to social work |  |
| Something related to sports |  |
| Something related to travelling |  |
| Something related to writing or directing |  |
| Something with children |  |
| Something with people |  |
| Stunt man |  |
| Tattoo artist |  |
| Teacher | ** |
| Teacher in elementary school |  |
| Teacher in handicraft |  |
| Teacher in high school |  |
| Teacher in language |  |
| Teacher in sports/PA |  |
| Theather technic |  |
| Translator |  |
| Waiter |  |
| Wheelchair diving instructor |  |
| Work in a theater |  |
| Work in business |  |
| Work in business law |  |
| Work in caféteria or bakery |  |
| Work in media |  |
| Work where I can travel and speek different languages |  |
| Work in a bank |  |
| Work in a restaurant |  |
| Writing |  |
| Youth worker for the church |  |
| Youtuber/vlogger |  |
| Note. The most frequent nominations are indicated with ** and several nominations with * | |
